# Hemophagocytic Lymphohistiocytosis Induced by Brucellosis: A Case Report

**DOI:** 10.7759/cureus.64287

**Published:** 2024-07-10

**Authors:** Daniel Park, Kevin Yoon, Amanda Lo, David Bolos

**Affiliations:** 1 Internal Medicine, University of California San Francisco Fresno, Fresno, USA; 2 Hematology and Oncology, Olive View - University of California Los Angeles (UCLA) Medical Center, Los Angeles, USA; 3 Pathology, Olive View - University of California Los Angeles (UCLA) Medical Center, Los Angeles, USA

**Keywords:** brucella melitensis, hyper-inflammatory syndrome, hlh-94, human brucellosis, hemophagocytic lymphohistiocytosis (hlh)

## Abstract

Hemophagocytic lymphohistiocytosis (HLH) is a hyper-inflammatory condition triggered by infections, malignancies, or autoimmune conditions. Brucellosis is a zoonotic disease contracted through exposure to infected animals or consumption of unpasteurized dairy products. The complications of both pathologies may be fatal. This report presents a rare instance of HLH induced by Brucellosis, highlighting the need for increased recognition of this life-threatening association.

## Introduction

HLH is a rare and severe inflammatory syndrome characterized by the dysregulated activation of macrophages and cytotoxic T-cells [1]. This pathologic process involves cytokine-mediated tissue injury and end-organ damage. The overwhelming inflammatory response results in heterogeneous clinical manifestations, including fevers, hepatosplenomegaly, liver injury, central nervous system (CNS) involvement, cytopenias, skin rashes, pleural and peritoneal effusions, and hemophagocytosis. HLH is classified as either primary or secondary. Primary (familial) HLH is caused by mutations affecting immune regulation and typically presents in children, whereas secondary (acquired) HLH is often triggered by infections, malignancy, or autoimmune disease. Predominantly, malignancy has been estimated to account for 45% of secondary HLH cases [2]. In instances of HLH induced by infection, Epstein-Barr virus (EBV) is the most common culprit. Commonly associated bacterial pathogens include *Mycobacterium tuberculosis* and *Rickettsia*. HLH secondary to brucellosis has been rarely reported.

Brucellosis is a zoonotic infection caused by various *Brucella *spp. that primarily affects wildlife and livestock [3]. It is often transmitted to humans through direct contact with infected organisms, with the primary known vector being unpasteurized milk. Brucellosis can infiltrate any organ system and has a diverse clinical presentation, with complications including endocarditis, osteoarticular complications, CNS dysfunction, pneumonia, and peritonitis. Due to its infiltration of the reticuloendothelial system, hepatosplenomegaly has been reported to occur in 63.3% of patients [4]. Additionally, cytopenias are common; in a study of 484 brucellosis patients, 21.5% had anemia, 18.8% thrombocytopenia, 14.6% leukopenia, and 5.8% pancytopenia [5]. We report a case of brucellosis-induced HLH to bring awareness of this unique pathology.

## Case presentation

A 60-year-old man with no prior medical history presented to the emergency department endorsing fevers, chills, night sweats, and a 50-to-20-pound weight loss for approximately three months. Concurrently, over a span of one to two weeks, he experienced a decreased appetite. Physical examination was notable for waxing and waning mentation and ecchymoses on his right lower extremity. Vital signs on admission demonstrated a temperature of 103.8°F, heart rate of 141 beats per minute (bpm), blood pressure of 135/75 mmHg, and an oxygen saturation of 95% on room air.

Initial laboratory evaluations included a complete blood count (CBC), which revealed white blood cells (WBC) at 4.6 K/cumm, neutrophils at 2.9 K/cumm, lymphocytes at 1.6 K/cumm, monocytes at 0.1 K/cumm, hemoglobin (Hgb) at 13.5 g/dL, and platelets at 53 K/cumm. Coagulation studies showed a prothrombin time (PT) of 15.4 seconds, an international normalized ratio (INR) of 1.26, and a partial thromboplastin time (PTT) of 56.6 seconds. The complete metabolic panel (CMP) was significant for an elevated creatinine of 1.52 mg/dL, aspartate aminotransferase (AST) of 370 U/L, alanine transaminase (ALT) of 82 U/L, total bilirubin (T. Bili) of 2.5 mg/dL, and direct bilirubin (D. Bili) of 1.0 mg/dL. Inflammatory markers were significantly elevated with ferritin >40,000 ng/mL, C-reactive protein (CRP) at 231.8 mg/L, and erythrocyte sedimentation rate (ESR) at 21 mm/hr.

**Figure 1:**
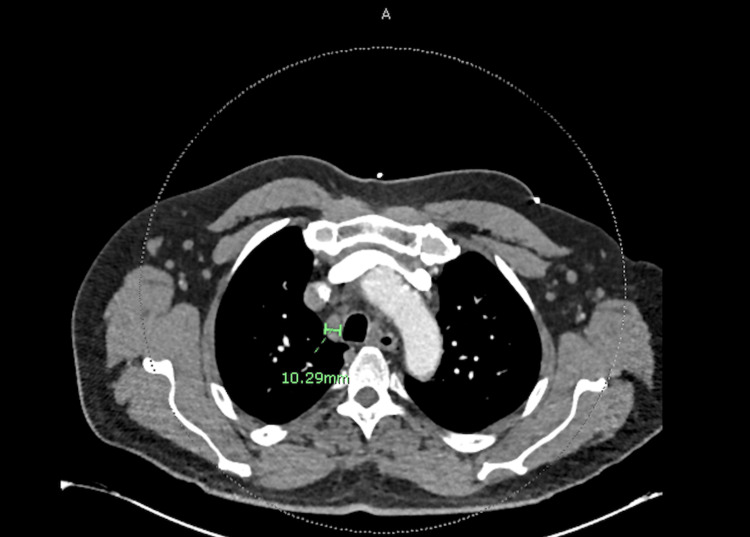
Cross section of contrast-enhanced CT scan showing 10.29 mm mediastinal lymph node

**Figure 2:**
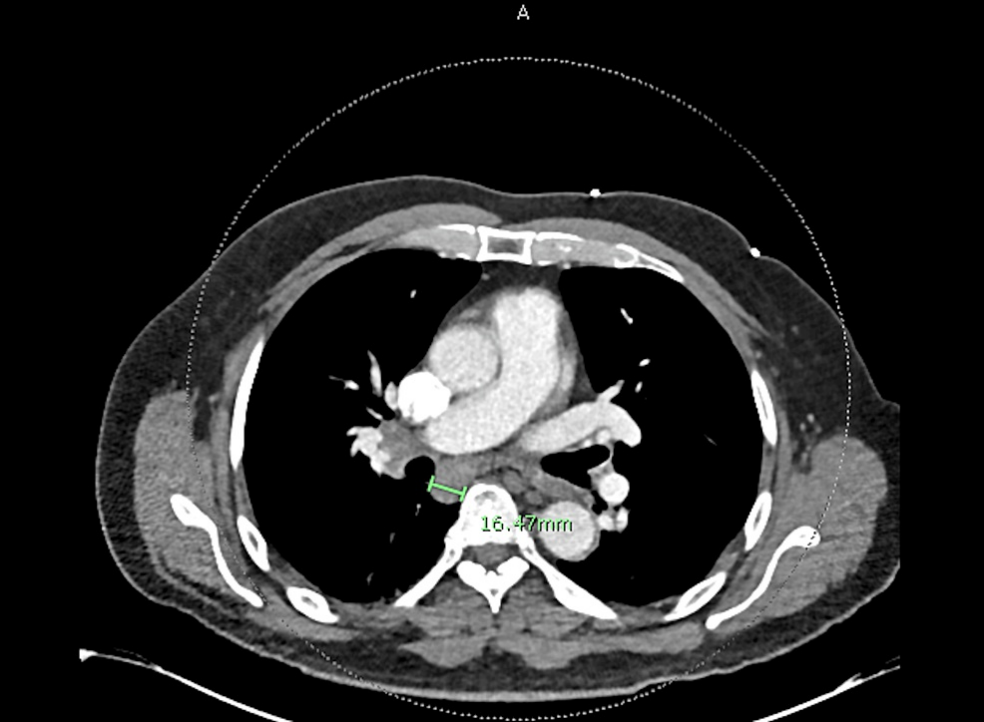
Cross section of contrast-enhanced CT scan showing 16.47 mm mediastinal lymph node

**Figure 3:**
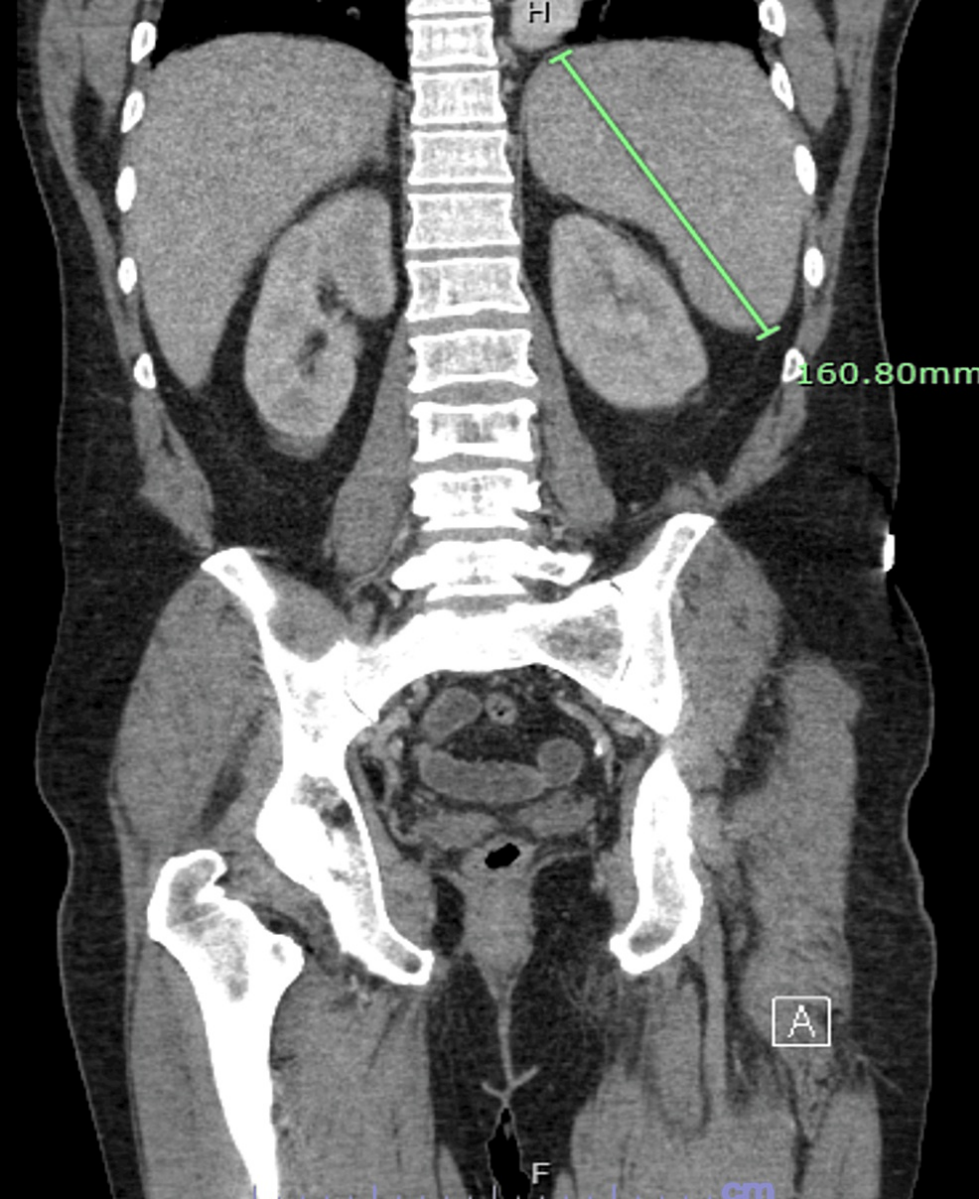
Cross section of contrast-enhanced CT scan showing splenomegaly, measuring approximately 16 cm

Radiographically, a computed tomography (CT) scan with contrast of his chest, abdomen, and pelvis was significant for multiple enlarged mediastinal and hilar lymph nodes (Figures 1, 2), including a 1.6 cm portocaval lymph node, splenomegaly measuring 16 cm, and prostatomegaly with heterogeneous enhancement of the prostate (Figure 3). His CT head showed no acute intracranial abnormality. CT lumbar spine with contrast and MRI lumbar spine with and without contrast also did not show any evidence of osteomyelitis. The transthoracic and transesophageal echocardiogram did not show any infectious vegetation.

**Figure 4:**
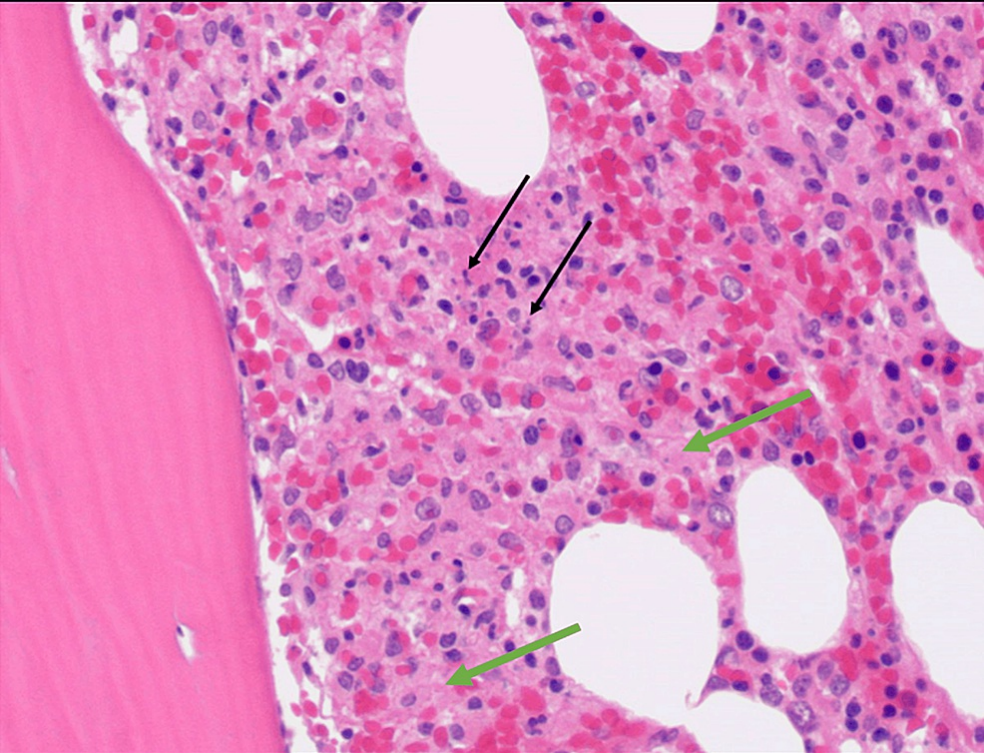
Bone marrow core biopsy showing increased background histiocytes (green arrow), including some containing phagocytosed cellular debris (black arrow). Hematoxylin and eosin stain, x100

**Figure 5:**
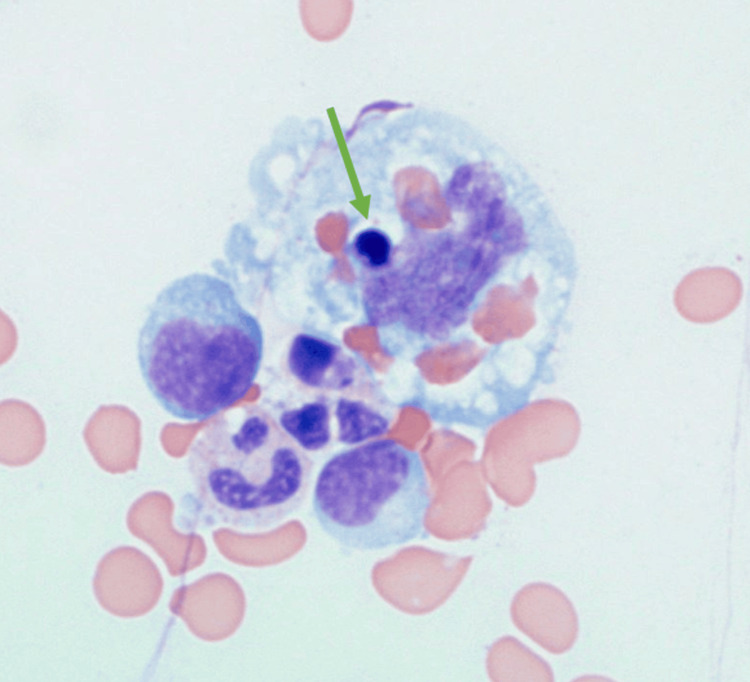
Bone marrow aspirate smear demonstrating hemophagocytic activity with engulfed erythrocytes and erythrocyte precursors (green arrow). Wright stain, x400

Further investigations during his hospital course were remarkable for blood cultures positive for *Brucella melitensis* growth, Brucella antibody agglutination >1:1280 (reference range: <1:80 titer), Brucella IgG at 2.08 (reference range: <0.80), Brucella IgM at 7.67 (reference range: <0.80), triglycerides at 335 mg/dL, fibrinogen at 85 mg/dL, soluble interleukin-2 receptor at 32,550 pg/mL (reference range: 532-1891 pg/mL), increased natural killer (NK) cell cytotoxicity at 80% dead (50:1 + IL-2, reference range ≥23% dead), and a bone marrow biopsy showing hemophagocytic histiocytes (Figures 4, 5).

On initial evaluation, the patient’s clinical status was undifferentiated, with a broad range of differential diagnoses including sepsis, meningitis, viral infection, and malignancy. Meningitis was deemed less probable as the patient did not endorse headache, neck stiffness, or photophobia. Furthermore, the radiographic findings of enlarged mediastinal and hilar lymph nodes and splenomegaly were inconsistent with the typical manifestations of meningitis. Infectious etiologies were investigated, with evaluations for human immunodeficiency virus, hepatitis, EBV, cytomegalovirus, parvovirus B19, Q fever, coccidioidomycosis, histoplasmosis, syphilis, and tuberculosis all resulting as negative.

It was suspected that there was an aspect of disseminated intravascular coagulation, reflected by his elevated PT, PTT, and INR, along with hypofibrinogenemia, elevated D-Dimer, and thrombocytopenia, which notably dropped to a nadir of 14 K/cumm. However, the underlying process contributing predominantly to both thrombocytopenia and hypofibrinogenemia was likely due to HLH.

Ultimately, HLH was diagnosed as the patient met at least five of the criteria outlined in the HLH-2004 protocol [2]. The criteria the patient met included: 1) fever >101.3°F; 2) splenomegaly; 3) hypertriglyceridemia >265 mg/dL and hypofibrinogenemia ≤1.5 g/dL; 4) hemophagocytosis in bone marrow; 5) serum ferritin concentration ≥500 µg/L; and 6) soluble IL-2 receptor ≥2400 U/mL. Although the patient had cytopenias affecting ≥2 of 3 lineages of the peripheral blood, besides thrombocytopenia <100×10^9^/L, he did not meet the HLH-2004 criteria of Hgb <9 g/dL and absolute neutrophil count <1.0×10^9^/L. Nevertheless, his clinical and serologic markers met more than five of the diagnostic criteria for HLH.

The patient received doxycycline 100 mg twice daily (BID) and rifampin 100 mg BID for six weeks as antimicrobial treatment for his brucellosis. In accordance with the HLH protocol's steroid regimen, the patient was started on dexamethasone 10 mg/m^2^ (20 mg I.V.). Despite his elevated ferritin persisting >40,000 ng/mL for two days post-steroid initiation, a significant response was observed on the third day, with the ferritin level dropping to 26,280 ng/mL, eventually decreasing to 4005 ng/mL prior to discharge. Thrombocytopenia and hypofibrinogenemia reached nadirs of 14 K/cumm and 85 mg/dL, respectively, and showed improvement with steroid therapy, with platelet counts rising to 123 K/cumm and fibrinogen to 193 mg/dL. Liver function tests also showed improvement, with a resolution of his transaminitis and a decrease in total bilirubin to 1.3 mg/dL and direct bilirubin to 0.5 mg/dL.

Clinically, the patient’s fluctuating mentation and undulating fevers resolved completely, with the patient reporting a return to his baseline health. The patient finished his steroid taper per the HLH taper recommendations.

## Discussion

HLH is a rare and potentially fatal hyperinflammatory syndrome characterized by immune dysregulation [6]. The aberrant activation of cytotoxic T-cells, natural killer cells, and macrophages results in a cytokine storm response, leading to subsequent immune-mediated injury to various organ systems. While the prevalence of secondary HLH is not well established, mortality rates are estimated to range from 20% to 88% [7]. The diverse array of triggers, such as malignancy, infections, therapy-related etiologies, and autoimmune disease, alongside the various manifestations such as fever, hepatosplenomegaly, lymphadenopathy, and cytopenias, renders HLH a diagnostic challenge.

HLH diagnosis requires five of eight HLH-2004 criteria: 1) fever; 2) splenomegaly; 3) cytopenias affecting ≥2 of 3 lineages of the peripheral blood (hemoglobin <9 g/dL, platelets <100×10^9^/L, neutrophils <1.0×10^9^/L); 4) hypertriglyceridemia and/or hypofibrinogenemia; 5) hemophagocytosis in the spleen, lymph nodes, or bone marrow; 6) low or no NK cell activity; 7) ferritin ≥500 µg/L; and 8) soluble IL-2 receptor ≥2400 U/mL (Table 1) [8]. Our patient fulfilled six of these criteria to be classified as HLH, and importantly, a trigger was identified through Brucella titers and blood cultures. Brucellosis, although a common zoonotic infection, is a rarely reported cause of secondary HLH. Brucellosis also has several overlapping characteristics with HLH, which poses an additional challenge as it has been known to cause cytopenias and hepatosplenomegaly through the involvement of the reticuloendothelial system [9].

**Table 1:** HLH-2004 diagnostic criteria Diagnosis of HLH is established if criteria 1 or 2 (5 of 8 criteria) are fulfilled HLH: hemophagocytic lymphohistiocytosis; NK: natural killer

		Criteria met by patient
Criteria 1	A molecular diagnosis consistent with HLH	
Criteria 2	Fever	X
	Splenomegaly	X
	Cytopenias affecting ≥2 of 3 lineages of the peripheral blood (hemoglobin <9 g/dL, platelets <100×10⁹/L, neutrophils <1.0×10⁹/L)	
	Hypertriglyceridemia and/or hypofibrinogenemia	X
	Hemophagocytosis in the spleen, lymph nodes, or bone marrow	X
	Low or no NK cell activity	
	Ferritin ≥500 µg/L	X
	Soluble IL-2 receptor ≥2400 U/mL	X

Identifying a trigger is crucial as mortality in adult HLH is primarily a result of the progression of the underlying trigger or refractory HLH [1]. Effective treatment depends on addressing the trigger. When treating HLH, the hyperinflammatory response is managed with the HLH-94 protocol, which includes dexamethasone, etoposide, cyclosporine, and intrathecal methotrexate [10]. However, immunosuppression may compromise the patient’s ability to defend against infectious pathogens. Prolonged immunosuppressive therapy also increases the risk of dormant infections reactivating and susceptibility to new pathogens. Therefore, appropriate antibiotic treatment is critical. Because the patient remained hemodynamically stable and relatively non-toxic, he was only managed with brucellosis treatment and dexamethasone alone. There are documented cases of HLH successfully managed solely by addressing the underlying cause. However, in cases of end-organ damage, adjunctive corticosteroids to control the cytokine storm have been recommended. HLH-induced Brucellosis in the literature has been shown to be managed in a spectrum of ways such as only with targeted antibiotics versus antibiotics plus a full HLH protocol regimen. Given the uniqueness of each patient and the nuance of clinical management, the current ASH guidelines are the most generalizable to patient care [11].

In refractory HLH, there is a paucity of data for second-line treatment. Case reports have been published describing the efficacy of infliximab, daclizumab, alemtuzumab, anakinra, and vincristine.

## Conclusions

HLH is a rare and potentially fatal inflammatory syndrome. While HLH is uncommon, its association with brucellosis is exceptionally unique. Clinicians should be aware of this atypical etiology when attempting to identify a trigger. Upon identification of brucellosis-induced HLH, prompt antibiotic therapy should be initiated. Additionally, in cases where end-organ damage is present, adjunctive corticosteroid therapy should be considered.
